# Books

**Published:** 1883-11

**Authors:** 


### Books.

A COMPEND OF CHEMISTRY. By G. Mason Ward, M.D., Demonstrator of Chemistry in Jefferson Med. College, Philadelphia, with Table of Elements. Philadelphia: P. Blakiston, Son & Co.

This compend claims no originality; is a mere quiz primer for classes in chemistry. It opens with an admirable tabular view of the elements, which enables the student at a glance, to ascertain their leading characteristics, their symbols, atomic weights, atomicity, specific gravity, discoverer, derivation of name, electrical condition, and the fusing point of the metals. We commend this little work to students and quiz-masters.

A GUIDE TO AMERICAN MEDICAL STUDENTS IN EUROPE. By Henry Huen, M.D., Lecturer on Diseases of the Nervous System in the Albany Medical College. New York: Wm. Wood & Co.

The great advantage, the urgent necessity for such a work as this is quite obvious. A large and increasing number of Americans go yearly to the great European centres of medicine and surgery, as students, many of them inexperienced even, in the ways of the world at home. How welcomed to the great majority of them, the timely revelations and directions of this guide of Dr. Huen's will be! Standing for the first time in some great German or French city, surrounded by a world of strangers, whose very language falls upon their ears like the babble of Babel; here, as if from a friendly couselor at the home fireside, they can obtain, in every emergency, just the information they need, and with only the trifling expense of the cost of the book.

The book seems to cover the entire ground; no place is omitted where students from America are accustomed to congregate for medical study or observation. In an appendix is given, even, all recent changes that have occurred in the corps of instructors of the different universities. We predict a steady demand for this little book by Dr. Huen.

---

THE MEDICAL STUDENTS' MANUAL OF CHEMISTRY. By R. A. Witthaus, A M., M.D, Prof. of Chemistry and Toxicology in the University of Buffalo, etc. New York: Wm. Wood & Co.

We are much pleased with this book. There are features in it which admirably adapt it to its purpose, the instruction of medical students; and such as are seldom found in similar works. It is sufficiently brief; its classifications are scientific, yet practical; its arrangement judicious, particularly that of placing organic chemistry immediately in connection with the treatment of carbon. The classification of the elements in accordance with their quantivalence and chemical characteristics, and not simply as metals and non-metals, is a decided improvement, though now generally adopted.

The treatment of the subject of acids, bases and salts, is more lucid and rational than most of the modern textbooks present that important subject of chemistry.

We have but one fault to find with the book. Why does the author persist in inverting the fixed order of chemical notation? He writes, for example, $CuSO_4$ thus: $SO_4Cu$, directly in the face of the rule, accepted by all good chemical authority, that the symbol of the positive constituent shall be written first, and after it that of the negative.

And then he is inconsistent with himself; in one place he writes $NO_3 H$, and in another KHO, yet both of these are tenary compounds. He has a reason for this deviation from the known rule, of course, but we see no force in it. This blemish as regards notation, appears with the same effect in the author's little work, entitled the Essentials of Chemistry; an excellent work, too, for its purpose.

---

THE TREATMENT OF WOUNDS; ITS PRINCIPLES AND PRACTICE, GENERAL AND SPECIAL By Lewis S Pilcher, A M., M D., Member of the New York Surgical Society. With 116 Wood Engravings. New York: Wm. Wood & Co. 1883.

This is number eighth of the famous Wood's Library of Standard Medical Authors. Section first of part first is devoted to the principles of wound treatment. Section third, or part second, treats of wounds of special regions.

This enterprising house has issued no work more useful in its field or more acceptable to the profession than this. It is fully abreast of the times in this branch of surgery; and we heartily commend the work to our students and the profession at large. The illustrations are numerous, and neatly and accurately executed. What especially strikes one in a close examination of the book is the plain, practical, lucid manner in which every principle and point is presented to the reader. This is an admirable hand-book for ready, quick consultation.

---

INSANITY CONSIDERED IN ITS MEDICO-LEGAL RELATIONS, "Ornare Res Ipsa Negat Contenta Soceri." By T. R. Buckham, A.M., M.D. Philadelphia: J. B Lippincott & Co. London: 16 South Hampton Street, Strand. 1883. This valuable work was received through the sterling house of S. P. Richards & Son, of Atlanta, Ga.

Review next number.

---

THE ROLLER BANDAGE. By Wm. Barton Hopkins, M.D., Surgeon of the Out Department of the Pennsylvania Episcopal and University Hospitals, etc. With 73 illustrations. Philadelphia: J. B Lippincott & Co. 1883. Received through the house of S. P. Richards & Son, Atlanta, Ga.

Notice next number.

